# DOSE-L1000-Viz: an interactive Shiny application for dose–response transcriptomic analysis, target-centric exploration, and signature search

**DOI:** 10.1093/bioinformatics/btaf353

**Published:** 2025-07-03

**Authors:** Junmin Wang

**Affiliations:** Data Sciences and Quantitative Biology, Discovery Sciences, Biopharmaceuticals R&D, AstraZeneca, Waltham, MA 02451, United States

## Abstract

**Summary:**

Understanding how small molecules perturb gene expression is critical for guiding drug discovery. We present DOSE-L1000-Viz, a Shiny application that facilitates comprehensive exploration of compound-induced transcriptomic responses across doses, time points, and cell types. Powered by a dose–response database, DOSE-L1000-Viz features interactive visualization, target-centric compound ranking based on efficacy and potency, and a signature search module using reference gene sets derived from generalized additive models. We benchmarked signatures derived from generalized additive models against traditional methods and demonstrated the utility of DOSE-L1000-Viz through use cases in transcription factor modulation and drug repurposing.

**Availability and implementation:**

DOSE-L1000-Viz and the backend data are publicly accessible at: https://dosel1000.com. All code is publicly hosted on GitHub (https://github.com/JmWangBio/DOSEL1000Viz) and archived via Zenodo (https://doi.org/10.5281/zenodo.15532392).

## 1 Introduction

The Library of Integrated Network-Based Cellular Signatures (LINCS) L1000 database is a valuable resource for studying how various compounds affect gene expression across a wide range of cell lines (Himmelstein *et al.* 2017, Subramanian *et al.* 2017). To support exploration of this high-dimensional dataset, several platforms have been developed. For example, L2S2, L1000CDS^2^, and signatureSearch support queries of gene signatures, while clue.io and L1000FWD extend this functionality with compound-centric visualizations of transcriptional responses (Duan *et al.* 2016, Subramanian *et al.* 2017, Wang *et al.* 2018, Duan *et al.* 2020, Marino *et al.* 2025).

Despite their utility, existing platforms share several limitations. First, many rely on the single-point analysis of gene expression measurements (Clark *et al.* 2014, Subramanian *et al.* 2017). By ignoring the dose–response aspect of the data, these approaches risk missing insights into concentration-dependent regulatory effects. This can lead to less reproducible gene signatures, reducing the reliability of downstream signature queries. Second, while gene signatures offer valuable system-level insights, identifying compounds that modulate the expression of individual therapeutic targets, such as transcription factors, remains a fundamental task in early drug discovery (Bushweller 2019). Many targets, despite their central roles in disease-relevant networks, are often considered “undruggable” due to the absence of ligand-binding pockets. As a result, modulating their expression indirectly—via upstream regulators—offers a viable alternative, as exemplified by BET inhibitors suppressing the expression of MYC (Bushweller 2019). Furthermore, identifying compounds that affect their transcription may reveal drug repurposing opportunities and motivate downstream investigation.

To overcome these limitations, we developed DOSE-L1000-Viz, an interactive Shiny application that supports (i) dose–response visualization, (ii) target-centric exploration, and (iii) signature search using gene signatures derived from dose–response models. The app is powered by our previously published DOSE-L1000 database, constructed by fitting over 140 million generalized additive models (GAMs) and robust linear models to the L1000 dataset (Wang and Novick 2023). This large-scale modeling not only produced more reliable estimates of transcriptional fold changes but also uncovered patterns of potency and efficacy across 33395 compounds, 978 genes, and 82 cell lines (see Section 3.1 for definitions of potency and efficacy). Building on this foundation, DOSE-L1000-Viz allows users to not only visualize dose response data but also rank compounds by log_2_ fold change, efficacy, or potency in modulating the expression of a selected target, tailored to user-selected cell lines and time points. In addition, DOSE-L1000-Viz features a module for querying user-defined gene sets against all signatures derived from GAMs in DOSE-L1000. We demonstrated the robustness of GAM-derived signatures by benchmarking them against signatures derived from the characteristic direction (CD) method (Duan *et al.* 2016). Together, these features make our platform a versatile tool for compound prioritization and hypothesis generation in drug discovery.

## 2 Methods

### 2.1 Backend data preparation

The magnitudes and *P*-values of compound-induced fold changes, along with the estimates and standard errors of potency and efficacy, were retrieved from the DOSE-L1000 database (Wang and Novick 2023). In brief, the authors applied a GAM-based approach to compute these values. Although sigmoidal curves find strong support in the field of biochemical kinetics (Wang, Isaacson, and Belta 2019, Kiwimagi *et al.* 2021), GAMs provide greater flexibility in capturing non-canonical dose–response relationships and reduce the risk of overfitting in regions with high expression variance (Hastie and Tibshirani 1999).

Dose response expression profiles and metadata, including compound identities, cell lines, treatment conditions, and genes were retrieved from the GEO datasets GSE92742 and GSE70138. Mechanism of action (MoA) annotations for compounds were retrieved programmatically using the L1000 API (https://clue.io/). All data were stored in a reference database implemented in SQLite, with tables indexed to ensure efficient querying (see Supplementary Fig. S1, available as supplementary data at *Bioinformatics* online for the database schema and Supplementary Table S1, available as supplementary data at *Bioinformatics* online for the database indexes).

### 2.2 Shiny app implementation

The app was built using the shiny R package (https://cran.r-project.org/package=shiny) (Chang *et al.* 2024). Plots in the app were generated using the ggplot2 package for static visualizations, which were then made interactive with the plotly package (Wickham 2016, Sievert 2020). Queries were performed using the RSQLite package (https://cran.r-project.org/package=RSQLite) (Müller 2001). The app and the database were hosted on a server running Ubuntu 24.04, with 4 vCPUs (Intel-based), 8 GB RAM, and 160 GB SSD storage. Deployment was managed using Shiny Server behind an Nginx reverse proxy to handle HTTP requests.

### 2.3 Signature similarity and statistical evaluation

To assess the reproducibility of gene signatures derived from different methods, we compared GAM-based and CD-based signatures across replicated perturbation conditions. GAM-based signatures were obtained from the DOSE-L1000 database, and CD-based signatures were downloaded from the L1000CDS^2^ portal (Duan *et al.* 2016, Wang and Novick 2023). For the GAM approach, gene signatures were defined as sets of significantly upregulated or downregulated genes (adjusted P<0.05 and |log_2_ fold change| > 1).

For each replicated condition defined by compound, cell line, time, and dose, we calculated the pairwise Jaccard index (i.e. overlap percentage) between batches to quantify overlap between gene signatures. To establish a baseline, we also generated 2000 random pairs of perturbation conditions across the dataset. For each random pair, Jaccard indices were calculated for both GAM and CD signatures, providing null distributions to represent similarity expected by chance.

In the “Signature Search” module, signature comparison was implemented using Fisher’s exact test. Further details can be found in the Supplementary Methods, available as supplementary data at *Bioinformatics* online.

## 3 Results

### 3.1 Visualization of dose–response, potency, and efficacy

DOSE-L1000-Viz enables users to interactively explore dose–response relationships for compound-gene pairs from the L1000 database. The “Dose Response Curves” tab supports up to six biological contexts (i.e. specific conditions defined by compound, gene, cell line, and time) and displays GAM-fitted dose–response curves.

**Figure 1.**
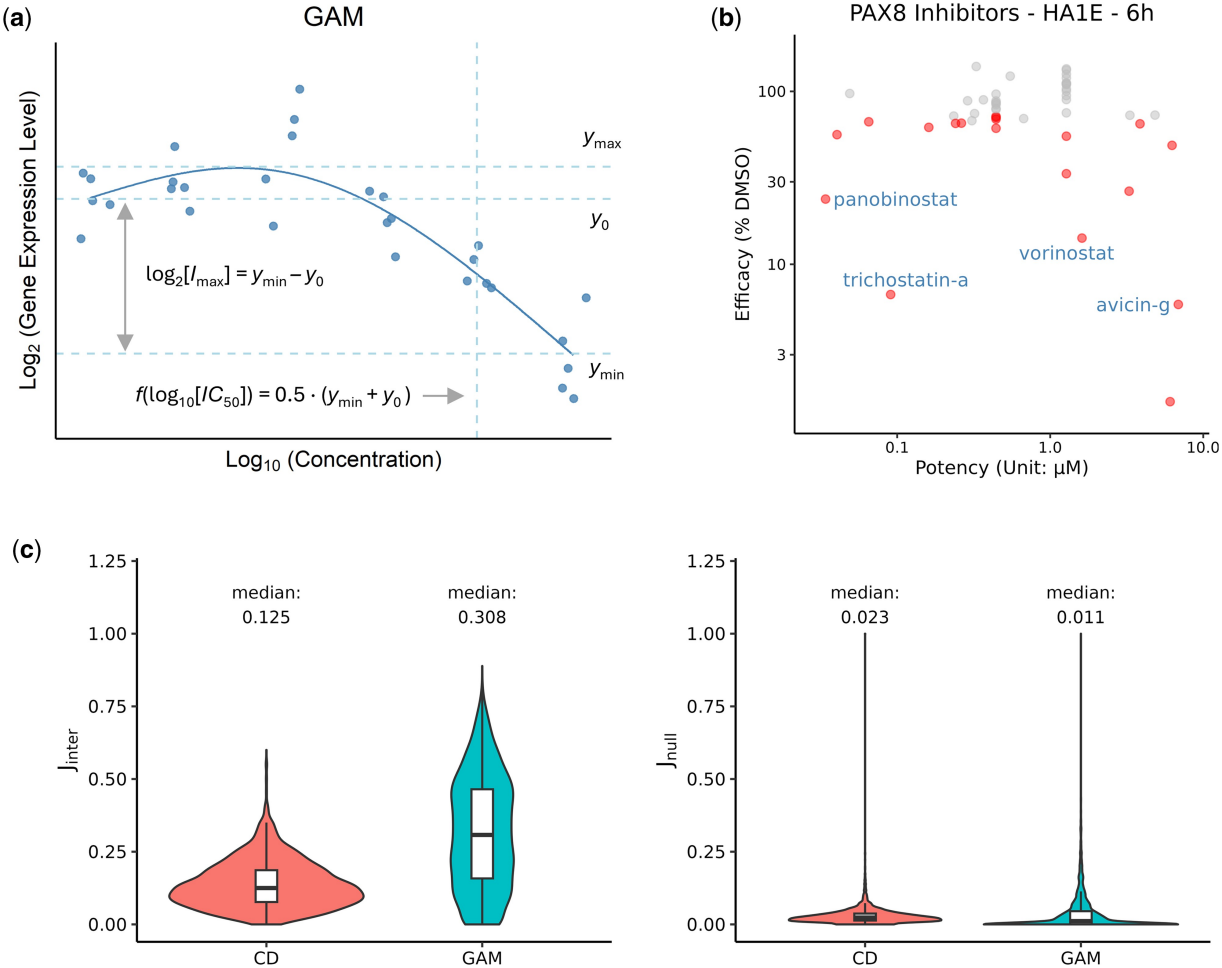
Overview of key features in DOSE-L1000-Viz and validation of GAM-derived signatures. (a) Definitions of potency and efficacy based on gene expression–dose response curves. (b) Top compounds predicted to suppress PAX8 expression in HA1E kidney cells six hours post treatment, ranked by efficacy. Compounds are highlighted if the 95% confidence interval of their efficacy estimate excludes 100% (i.e. no change in expression). Known HDAC inhibitors are labeled. (c) Distribution of inter-batch Jaccard indices for GAM- and CD-derived gene sets compared to null distributions generated from random pairs of conditions.

To compare the efficacy and potency of compound-gene pairs, users can generate scatter plots with error bars representing 95% confidence intervals in the “Efficacy versus Potency” tab. Each compound was classified as a gene-specific activator or inhibitor by comparing maximum and minimum log_2_ fold changes relative to the vehicle control. Efficacy was defined as the maximum log_2_ fold change: $\log_{2}\left[I_{\max}\right]$ (maximum decrease) for inhibitors, and $\log_{2}\left[E_{\max}\right]$ (maximum increase) for activators (Fig. 1a). Potency was defined as the log_10_-transformed concentration yielding a half-maximal effect: $\log_{10}\left[{IC}_{50}\right]$ for inhibitors and $\log_{10}\left[{EC}_{50}\right]$ for activators (Fig. 1a) (Wang and Novick 2023). This visualization facilitates comparison of gene regulation strength and sensitivity across different compounds, genes, or cell lines.

### 3.2 Target-centric exploration of compound effects

The “Target View” tab provides a target-centric workflow that enables users to identify compounds that modulate the expression of a target gene. For each selected context, compounds can be ranked by their estimated efficacy and potency in upregulating or downregulating the target.

To illustrate this functionality, we applied it to a specific use case for PAX8, a transcription factor frequently dysregulated in kidney cancer (Barr *et al.* 2015). Though PAX8 is considered undruggable, targeting its upstream regulators may present a viable strategy for indirect inhibition. Using our app, we ranked compounds by their efficacy in suppressing PAX8 expression in HA1E cells—a premalignant, SV40-transformed kidney-derived cell line—six hours post treatment (Lu *et al.* 2004). Among the top-ranked compounds were several HDAC inhibitors, including trichostatin A, vorinostat, and panobinostat, confirming previous accounts of HDACs’ role in PAX8 regulation (Fig. 1b) (Di Palma and Zannini 2022). Furthermore, we identified avicin G, a triterpenoid compound that inhibits NF-κB signaling, as a potential PAX8-suppressing agent (Fig. 1b) (Haridas *et al.* 2001). While notably less potent than HDAC inhibitors, its distinct mechanism suggests an alternative regulatory pathway that may merit further investigation.

In the “Target View” tab, users can also rank compounds by the *P*-value of their log_2_ fold change at individual doses. The “Compound View” tab provides detailed profiles for each compound.

### 3.3 GAM-derived signature search

We also implemented a “Signature Search” module that allows users to compare input gene sets against 82741 GAM-derived signatures in the DOSE-L1000 database using Fisher’s exact test. To evaluate the robustness of GAM-derived signatures, we identified 1403 compound-cell line-time-dose conditions that were profiled in at least two independent batches. Across these conditions, signatures derived from GAMs demonstrated consistently higher reproducibility than those derived from the CD method. The distribution of Jaccard indices was further shifted to the right for GAMs, with a median value of 0.308 compared to 0.125 for CD-based gene sets (Fig. 1c). In contrast, random comparisons across unrelated conditions yielded near-zero Jaccard indices for both methods, confirming that neither method inherently produces higher similarity (Fig. 1c). These findings support the markedly superior robustness of GAM-derived signatures, enhancing their reliability for downstream applications.

To illustrate the functionality of this module, we queried the gene signature of pravastatin, an HMGCR inhibitor, in MCF7 breast cancer cells. The query took approximately three minutes to complete. Among the top-ranked matches were several HDAC inhibitors, including apicidin and trichostatin A. These compounds shared transcriptional effects with pravastatin, such as downregulation of NRIP1, a gene implicated in chromatin remodeling (Wei *et al.* 2000). This overlap suggests that pravastatin may exert HDAC-inhibitory activity in this context, revealing a potential off-target mechanism with implications for drug repurposing in breast cancer.

With all functionalities, the queried data can be downloaded for further analysis or integration with external tools. In addition, the app includes a “ReadMe” tab that provides detailed instructions and explanations, complete with examples to guide users in navigating the various functionalities. Users can also download the complete backend data as individual RDS files directly through the app. For those interested in deploying the database internally, the code to reconstruct the SQLite database from RDS files is available in the GitHub repository mentioned in the abstract.

## 4 Conclusion

In this paper, we introduced DOSE-L1000-Viz, a Shiny-based application that facilitates dose-aware exploration of compound-induced transcriptional responses, with broad applications in drug discovery and repurposing. A key feature of our platform is its target-centric exploration module. This module could not only inform indirect modulation strategies for undruggable targets, as demonstrated, but also aid in hit prioritization based on efficacy and potency. Compounds with the strongest potency against a target may serve as leads for further optimization.

We also introduced and validated a signature search module powered by GAM-derived transcriptional signatures. This module provides systematic insights into shared MoA or unexpected off-target activities. A compound not previously associated with a particular mechanism may emerge as relevant, opening new avenues for its application.

Despite these advances, several limitations remain. First, GAM-derived signatures require multi-dose transcriptomics data, limiting their applicability to datasets with sparse dose coverage. Second, our platform is restricted to the 978 landmark genes profiled in the L1000 assay. Finally, our current implementation of signature search relies on comparison of gene identities. Future work may explore integrating gene embeddings into signature search for improved context-awareness (Chen *et al.* 2024).

## Supplementary Material

btaf353_Supplementary_Data

## Data Availability

DOSE-L1000-Viz and the backend data are publicly accessible at: https://dosel1000.com. All code and data are archived via Zenodo (https://doi.org/10.5281/zenodo.15532392).
