# Supplementary material for: DOSE-L1000-Viz: an interactive Shiny application for dose–response transcriptomic analysis, target-centric exploration, and signature search
Source: Bioinformatics. 2025 Jul 3;41(7):btaf353. doi: 10.1093/bioinformatics/btaf353 (PMC12237504; doi:10.1093/bioinformatics/btaf353)
Supplement: btaf353_Supplementary_Data [file btaf353_supplementary_data.pdf]

DOSE-L1000-Viz: An Interactive Shiny Application for Dose-Response Transcriptomic Analysis, Target-Centric Exploration, and Signature Search

Junmin Wang[1,*]

[1] *Data Sciences and Quantitative Biology, Discovery Sciences, Biopharmaceuticals R&D, AstraZeneca, Waltham, Massachusetts*

**Corresponding author: Junmin Wang (jmwang.bio@gmail.com)*

Supplementary Information

- Supplementary Methods
- Supplementary Tables
- Supplementary Figures
- Supplementary References

Supplementary Methods

Generation of Reference Signatures

GAM-derived signatures were defined as sets of significantly upregulated and downregulated genes identified through generalized additive model (GAM) fitting, as described previously (Wang and Novick 2023). A gene was considered significant if it met the thresholds of adjusted $p < 0.05$ and $|\log_2$ fold change$| > 1$. A reference signature was retained for inclusion in the searchable database if it contained at least one significantly upregulated or downregulated gene. Using this criterion, we compiled a total of 82741 reference signatures across all conditions in the DOSE-L1000 database.

Similarity Metric for Signature Comparison

To quantify similarity between a pair of signatures, we considered the Jaccard Index (i.e., overlap percentage), defined as the ratio between the number of overlapping genes and the total number of unique genes in the union of two signatures:

$$\text{Jaccard}(A, B) = \frac{|A \cap B|}{|A \cup B|}$$

where *A* and *B* are the sets of genes in the two signatures.

Signature Search Using Fisher’s Exact Test

For each user-provided query, we compared the submitted upregulated and downregulated gene sets separately against the corresponding up and down gene sets from all 82741 reference signatures in the DOSE-L1000 database. Specifically:

- The user’s upregulated gene set was compared against the upregulated genes of each reference signature.
- The user’s downregulated gene set was compared against the downregulated genes of each reference signature.

For each comparison, a Fisher’s exact test was used to assess whether the overlap between the query and reference gene sets was greater than expected by chance. The resulting p-values were adjusted for multiple testing using the Benjamini-Hochberg (BH) method. A reference signature was considered significant if adjusted p-value of either the upregulated or downregulated comparison was less than 0.05.

Supplementary Tables

**Index Name**	**Table Name**	**Indexed Columns**
idx_c_broadiXcelli	condition	broad_id, cell_id
idx_c_celli	condition	cell_id
idx_c_broadbatchiXcelli	condition	broad_batch_id, cell_id
idx_gi_s	gene_info	symbol
idx_p_cn	pert	cpd_name
idx_cg_condiXg	condition_gene	cond_id, gene
idx_cg_g	condition_gene	gene
idx_t_cgiXpd	test	cond_gene_id, pert_dose
idx_drc_cgi	drc	cond_gene_id
idx_dmso_pliXg	dmso	plate_id, gene

Supplementary Table 1. Overview of database indexes, including indexed tables and columns**.**

Supplementary Figures

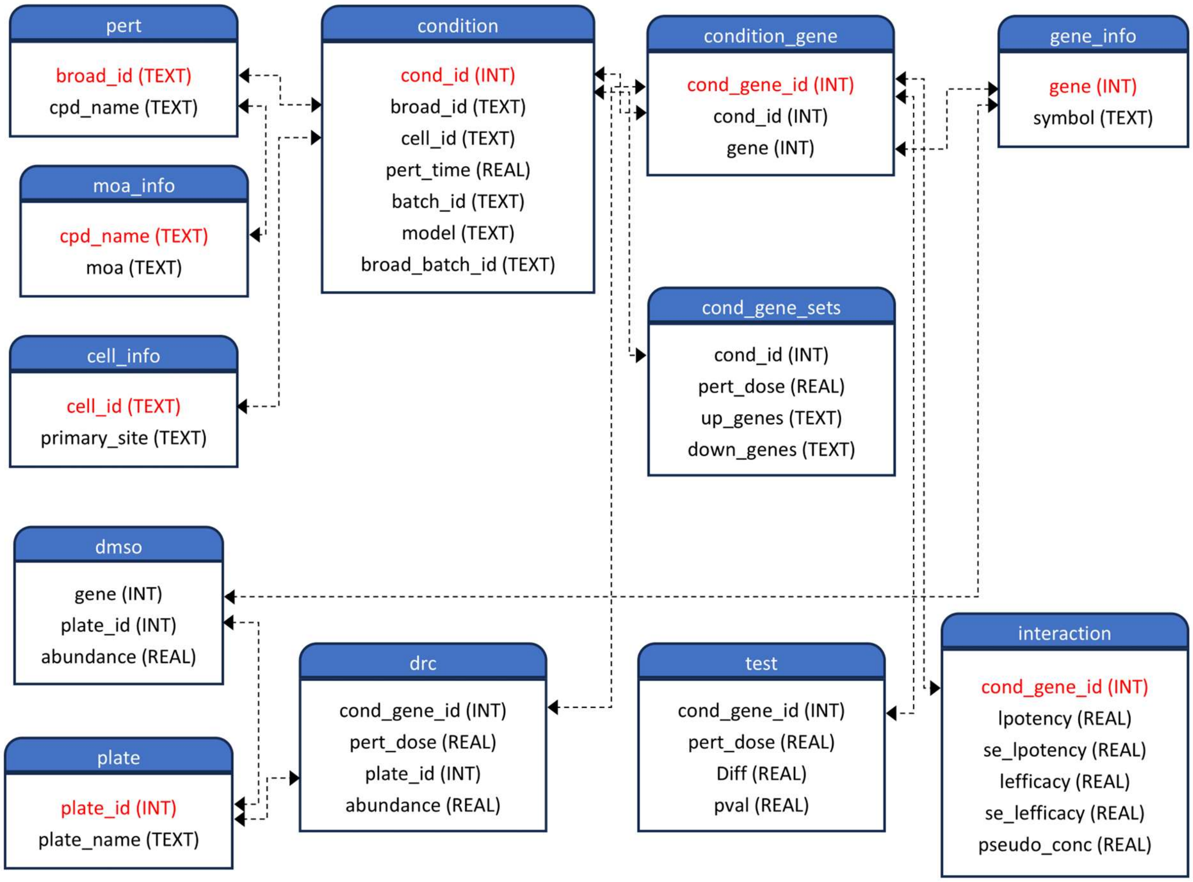

Supplementary Fig. 1. **Schema of the backend database**. Tables, fields within each table, and their respective data types are displayed. Primary key fields are highlighted in red. Dashed arrows represent the relationships between fields across different tables that are joined during queries.

Supplementary References

Wang, J., and S. Novick. 2023. 'DOSE-L1000: unveiling the intricate landscape of compound-induced transcriptional changes', *Bioinformatics*, 39.
